# Prevalence of Psychological Distress and Its Risk Factors in Patients with Primary Bone and Soft Tissue Tumors

**DOI:** 10.3390/healthcare9050566

**Published:** 2021-05-11

**Authors:** Masato Ise, Eiji Nakata, Yoshimi Katayama, Masanori Hamada, Toshiyuki Kunisada, Tomohiro Fujiwara, Ryuichi Nakahara, Shouta Takihira, Kohei Sato, Yoshiteru Akezaki, Masuo Senda, Toshifumi Ozaki

**Affiliations:** 1 Department of Orthopaedic Surgery, Okayama University Hospital, Okayama 700-8558, Japan; masato.sei@gmail.com (M.I.); toshikunisada@gmail.com (T.K.); tomomedvn@gmail.com (T.F.); pikumin55@gmail.co.jp (R.N.); seikei_saikou@yahoo.co.jp (S.T.); kohei.sato117@gmail.com (K.S.); tozaki@md.okayama-u.ac.jp (T.O.); 2 Department of Rehabilitation Medicine, Okayama University Hospital, Okayama 700-8558, Japan; yoshimikatayama@yahoo.co.jp (Y.K.); pjvy4uc1@okayama-u.ac.jp (M.H.); senda@md.okayama-u.ac.jp (M.S.); 3 Division of Physical Therapy, Kochi Professional University of Rehabilitation, Kochi 781-1102, Japan; akezakiteru@yahoo.co.jp

**Keywords:** psychological distress, distress and impact thermometer, bone and soft tissue tumor, surgery

## Abstract

Psychological distress is common in patients with soft tissue and bone tumors. We first investigated its frequency and the associated risk factors in patients with pre-operative bone and soft tissue tumors. Participants included 298 patients with bone and soft tissue tumors who underwent surgery in our institution between 2015 and 2020. Psychological distress was evaluated by the Distress and Impact Thermometer (DIT) that consists of two types of questions (questions about the severity of the patient’s distress (DIT-D) and its impact (DIT-I)). We used a cut-off point of 4 on the DIT-D and 3 on the DIT-I for screening patients with psychological distress. We therefore investigated: (1) the prevalence of psychological distress as assessed with DIT or distress thermometer (DT), which can be decided by DIT-D ≥ 4, (2) what are the risk factors for the prevalence of psychological distress, and (3) what is the number of patients who consulted a psychiatrist for psychological distress in patients with pre-operative bone and soft tissue tumors. With DIT and DT, we identified 64 patients (21%) and 95 patients (32%), respectively, with psychological distress. Multivariate logistic regression revealed that older age, sex (female), malignancy (malignant or intermediate tumor), a lower Barthel Index, and higher numeric rating scale were risk factors for psychological distress. Two patients (3%) consulted a psychiatrist after surgery. In conclusion, careful attention to psychological distress is needed, especially for female patients, older patients, and those with malignant soft or bone tissue tumors who have more than moderate pain.

## 1. Introduction

Psychological distress is common in patients with cancer. Approximately 22–66% of them experience psychological distress at some time during the course of the disease [1,2]. Psychological distress frequently impairs quality of life of patients and has a negative effect on survival [1,2]. However, distress tends to be undetected and therefore untreated by oncologists in daily practice [1,2]. Therefore, an easier-to-use screening tool is desired to distinguish between patients with clinically significant versus normal levels of psychological distress.

Although a number of measures have been used, such as the Hospital Anxiety and Depression Scale (HADS), distress thermometer (DT), and Distress and Impact Thermometer (DIT), definitive conclusions cannot be drawn regarding which tool is the best for distress screening due to the lack of randomized controlled trials comparing these tools [1,2,3]. The DIT was developed by adding an impact component (DIT-I) to the DT to screen for psychological distress more precisely [3,4,5,6,7]. The DIT consists of two types of items: questions about the severity of the patient’s distress (DIT-D) and its impact (DIT-I). A combination of the DIT-D and DIT-I may show higher specificity with preserved sensitivity in screening for clinically significant psychological distress compared to using the DT alone [4]. Previously, we showed that upper limb function was significantly associated with psychological distress evaluated by the DIT in breast cancer patients with axillary lymph node dissection [8].

Soft tissue and bone tumors are heterogeneous with various types of histology [9]. Several reports have been published about psychological distress in patients with malignant bone and soft tissue tumors, and 13–63% of them experience psychological distress [10,11,12,13,14,15,16,17,18]. However, data have been limited to outcomes of patients with various treatments and stages of them. To the best of our knowledge, no studies have focused on the DIT in the pre-operative period in patients with primary malignant bone and soft tissue tumors without metastasis as well as those with benign tumors. Moreover, no studies have evaluated potential risk factors that are correlated with psychological distress in patients with pre-operative primary soft tissue and bone tumors. We therefore investigated: (1) the prevalence of psychological distress as assessed with DIT or distress thermometer (DT), which can be decided by DIT-D ≥ 4, (2) what are the risk factors for the prevalence of psychological distress, and (3) what is the number of patients who consulted a psychiatrist for psychological distress in patients with pre-operative bone and soft tissue tumors.

## 2. Methods

### 2.1. Patients and Methods

We retrospectively evaluated the medical records of patients with primary bone and soft tissue tumors who underwent surgical resection in our institution between September 2015 and March 2020. Inclusion criteria were histological diagnosis of primary bone or soft tissue tumor, age ≥ 18 years, no metastasis, and no medical history of psychiatric disorders. We excluded patients with hematologic malignancy, bone metastasis from cancer, and those who had previously undergone surgery for the same lesion and non-curative resection. Then, a total of 298 patients (172 men and 126 women) were included in this study (Table 1). The median age was 53 years (range, 18–87 years). Cases included 54 bone tumors with 21 malignant tumors, 15 intermediate tumors, and 18 benign tumors, as well as 244 soft tissue tumors with 84 malignant tumors, 16 intermediate tumors, and 144 benign tumors. In Japan, there is a registry system of bone and soft tissue tumors that is managed by the Japanese Orthopaedic Association, in which about 1200 patients were registered per year [19,20]. Our institution (Okayama University Hospital) is a high-volume center, in which about 170 patients of bone and soft tissue tumors received surgery per year. Chemotherapy was performed in 20 patients, and radiotherapy was performed in three patients before surgery. Their disease treatment, and prognosis were explained to all patients before admission to the hospital. Benign bone and soft tissue tumors have a low recurrence rate (0–20%) and no metastasis after surgery. On the other hand, wide resection results in postoperative dysfunction of upper and lower limb in malignant bone and soft tissue tumors. In addition, local recurrence and metastasis may occur in 10–30% [9].

### 2.2. Assessment of Psychological Distress

In our institution, we routinely assess psychological distress with the DIT in every patient at the date of admission to the hospital. All patients were asked to complete the DIT at the time of admission to the Orthopedic Ward for surgery. They were asked whether they hoped to consult a psychiatrist, and each patient who hoped to consult a psychiatirist was able to do so. The DIT is a self-reported thermometer as a screening tool; sensitivity and specificity are both 0.82 [3]. The DIT has been validated by HADS, which is one of the most validated screening tools [3,4,6]. The reliability and usefulness of the DIT for investigating psychological distress have been shown in various cancers [4,5,6,7,8]. Baken showed that the combination of the DIT-D and DIT-I showed almost the same sensitivity (81% vs. 85%) and better specificity (82% vs. 66%) than the DT in patients with various cancers for detecting anxiety and distress by using HADS as the gold standard [4]. Itani investigated the reliability of the DIT for screening for psychological distress in newly diagnosed gynecological cancer patients by using HADS as the gold standard [6]. He reported the reliability of the DIT, which had sensitivity, specificity, positive predictive value, and negative predictive values of 0.893, 0.825, 0.781, and 0.917, respectively.

The DIT has a 0–10 scale, with 0 indicating no distress and 10 indicating extreme distress. The DIT consists of two types of items: questions about the severity of the patient’s distress (DIT-D) and its impact (DIT-I). Patients are instructed to choose the number (0–10) that best describes how much distress they have been experiencing in the previous week for DIT-D, and patients are also instructed to choose the number (0–10) that best describes how much impact the distress has on their daily life activity for DIT-I. The standard cut-off scores of the DIT for screening psychological distress are as follows: for adjustment disorders, a DIT-D score of 4 or above (the same validated cut-off score used by the DT, which is also recommended by the National Comprehensive Cancer Network (NCCN) guidelines for screening for psychological distress [21] and a DIT-I score of 3 or above; for depression, a DIT-D score of 5 or above and a DIT-I score of 4 or above; and for major depression with suicidal ideation, a DIT-D score of 5 or above and a DIT-I score of 5 or above. In this study, we used a cut-off point of 4 on the DIT-D and 3 on the DIT-I for screening patients with psychological distress, as reported previously [3]. We divided the patients into two groups: distress group (DIT-D ≥ 4 and DIT-I ≥ 3) and no distress group (those with a score below the cut-off). We assessed the prevalence of psychological distress with DIT and compared the prevalence of psychological distress assessed with DT, which can be decided by DIT-D ≥ 4. Furthermore, we assessed the number of patients who consulted a psychiatrist.

### 2.3. Risk Factors for Psychological Distress

To assess risk factors for psychological distress, clinical data at admission were assessed the day before surgery: median 2 days (range, 1–7 days), including demographics (age, sex, body mass index), socio-economic characteristics (smoking and drinking history, marital status, employment status living with a housemate, and having children), clinical characteristics (malignancy, tumor site, pain, neoadjuvant chemotherapy, and pre-operative radiotherapy), and physical function and activities of daily living status (Eastern Cooperative Oncology Group Performance Status and Barthel Index). Pain at the tumor site during motion was assessed with the numeric rating scale (NRS). This patient-based assessment tool evaluates the pain intensity on a scale of 0 (no pain) to 10 (worst pain) [22]. The level of pain (NRS) was decided based on the categorical pain scale of the NCCN guidelines: none (0), mild (1–3), moderate (4–6), or severe (7–10) [22]. Eastern Cooperative Oncology Group Performance Status (ECOG PS), which is a score ranging from zero (fully active) through three (capable of only limited self-care) to five (dead) [23]. The Barthel Index is a measuring tool of the individual’s performance on 10 activity of daily living functions. Additionally, it is scored in five-point increments, giving a score of 0–100 [24].

### 2.4. Statistical Analyses

Univariate analysis was performed to examine the associated factors that were statistically significant between the distress group and non-distress group. The Student’s *t*-test, χ2 test, and Fisher’s exact test were used to calculate the significance of demographic differences and clinical variables in the two groups. In addition, to examine the factors associated with screening for psychological distress, we performed multivariable logistic regression for items that were statistically significant in univariate analysis. Statistical analyses were performed using Bell Curve for Excel ver. 3.21 (Social Survey Research Information Co., Tokyo, Japan). *p* < 0.05 was considered statistically significant, and all tests were two-sided.

## 3. Results

### 3.1. The Factors Associated with Psychological Distress and Their Frequency in Pre-Operative Patients with Bone and Soft Tissue Tumors

In all patients, the median DIT-D and DIT-I scores were 2.3 (range: 0 to 10) and 1.5 (range: 0 to 10), respectively. Ninety-five patients (32%) scored ≥4 on the DIT-D, whereas 203 patients (68%) reported a score of <4. Eighty-two patients (28%) scored ≥3 on the DIT-I, whereas 216 patients (72%) reported a score of <3. Thus, 64 patients (21%) were identified as having psychological distress (score ≥ 4 on DIT-D and ≥3 on DIT-I) according to the DIT. Psychological distress was identified in 32%, 19%, and 15% of the patients with malignant, intermediate, and benign bone and soft tissue tumors, respectively. In patients with bone tumors, 15 patients (28%) were identified as having psychological distress. Psychological distress was identified in 10 (48%), 5 (33%), and 0 (0%) patients with malignant, intermediate, and benign bone tumors, respectively. Among patients with malignant bone tumors, psychological distress was identified patients with osteosarcoma (50%), and chondrosarcoma (G2) (40%). Among patients with intermediate bone tumors, psychological distress was identified patients with giant cell tumor (40%) and there are no patients with psychological distress in benign bone tumors (Table 2).

In patients with soft tissue tumors, 49 patients (20%) were identified as having psychological distress. Psychological distress was identified in 24 (28%), 1 (6%), and 24 (17%) patients with malignant, intermediate, and benign soft tissue tumors, respectively.

Among malignant soft tissue tumors, psychological distress was identified in many patients with liposarcoma (27%), and undifferentiated sarcoma (32%). Among patients with benign soft tissue tumors, psychological distress was identified in many patients with schwannomas (26%), which arise from Schwann cells of the peripheral nerve sheath. However, it was identified in few patients with lipomas, a slow growing, fatty lump most often arise in subcutaneous tissue and intra-muscle (12%) and tenosynovial giant cell tumors, which arise from synovial tissue of joint, bursae, and tendon sheaths of patients under 40 years old and sometimes cause pain by infiltrating surrounding tissues. (13%) (Table 3).

When we investigated psychological distress with DT, which can be decided by DIT-D ≥ 4, it was identified in 95 patients (32%) among all patients. Psychological distress was identified in 40 (25%), 9 (28%), and 46 (44%) patients with malignant, intermediate, and benign bone and soft tissue tumors, respectively. In patients with bone tumors, the number of DT-positive patients was 14 (67%), 5 (33%), and 4 (22%) in those with malignant, intermediate, and benign bone tumors, respectively. In patients with soft tissue tumors, the number of DT-positive patients was 32 (38%), 4 (24%), and 36 (25%) in those with malignant, intermediate, and benign bone tumors, respectively.

Univariate analysis revealed that older age, sex (female), malignancy (malignant or intermediate tumor), higher NRS, lower Barthel index, and performance status were factors related to distress (Table 4). Multivariable logistic regression analysis revealed that older age, sex (female), malignancy (malignant or intermediate tumor), lower Barthel Index, and higher NRS were risk factors for psychological distress. Psychological distress was identified in 29% of patients with malignant or intermediate tumors and in 15% of patients with benign tumors (odds ratio = 2.15, 95% confidence interval 1.13–4.08, *p* < 0.05). Psychological distress was identified in 17% of male patients and in 27% of female patients (*p* < 0.05). We further evaluated the association between the level of pain and the DIT. Psychological distress was identified in 33 of 96 patients (34%) with moderate or severe pain (NRS ≥ 4) and in 31 of 202 patients (15%) with no or mild pain (NRS < 4) (*p* < 0.01).

### 3.2. Patients Treated by a Psychiatrist

Only two patients hoped to consult a psychiatrist after surgery. This rate was 0.7%, and 3% of all patients and those who had psychological distress as assessed by DIT, respectively. Both patients had a malignancy (one osteosarcoma and one epithelioid sarcoma) with moderate pain and had psychological distress as assessed by DIT. They were able to consult a psychiatrist and underwent treatment.

### 3.3. Factors Associated with Psychological Distress and Their Frequency in Pre-Operative Patients with Malignant Bone and Soft Tissue Tumors

In patients with malignant bone and soft tissue tumors, the median DIT-D and DIT-I scores were 3.1 (range: 0 to 10) and 2.1 (range: 0 to 10), respectively. Forty-six patients (44%) scored ≥ 4 on the DIT-D, whereas 59 patients (56%) reported a score of <4. Forty-one patients (39%) scored ≥3 on the DIT-I, whereas 64 patients (61%) reported a score of <3. Thirty-four patients (32%) were identified as having psychological distress (score ≥ 4 on DIT-D and ≥3 on DIT-I) according to the DIT. Univariate analysis revealed that sex (female), neoadjuvant chemotherapy, smoking, and alcohol consumption were factors related to psychological distress (Table 5). Multivariable analysis revealed that not receiving neoadjuvant chemotherapy was a risk factor for psychological distress. Psychological distress was identified in 26% of patients not receiving neoadjuvant chemotherapy and 6% of patients who received neoadjuvant chemotherapy (odds ratio = 6.07, 95% confidence interval 1.22–30.1, *p* < 0.05).

### 3.4. Factors Associated with Psychological Distress and Their Frequency in Pre-Operative Patients with Benign Bone and Soft Tissue Tumors

In patients with benign bone and soft tissue tumors, the median DIT-D and DIT-I scores were 1.9 (range: 0 to 10) and 1.2 (range: 0 to 10), respectively. Forty patients (25%) scored ≥4 on the DIT-D, whereas 121 patients (75%) reported a score of <4. Thirty-one patients (19%) scored ≥3 on the DIT-I, whereas 130 patients (81%) reported a score of <3. Twenty-four patients (15%) were identified as having psychological distress according to the DIT. Psychological distress was identified in no patients with benign bone tumors, and in 17% of patients with benign soft tissue tumors. Univariate analysis revealed that older age and higher NRS were factors related to psychological distress (Table 6). Multivariable analysis revealed that older age and higher NRS were risk factors for psychological distress.

We further evaluated the association between pain level and the DIT. Psychological distress was identified in 18 of 51 patients (35%) with moderate or severe pain (NRS ≥ 4), and in 6 of 110 patients (5%) with no or mild pain (NRS < 4) (*p* < 0.01).

## 4. Discussion

### 4.1. Psychological Distress in Bone and Soft Tissue Tumor Patients

A few reports have described psychological distress in pre-operative cancer patients; 6–19% of such patients experience distress [25,26]. To the best of our knowledge, only one report has focused on psychological distress in the pre-operative period in malignant bone and soft tissue tumor patients [15]. Tang reported psychological distress in 13% of patients with malignant bone and soft tissue tumors using the Depression, Anxiety, and Stress Scale—21 Items [17]. However, they included 25% of patients with a past history of depression and/or anxiety, which would have a significant effect on evaluating the present psychological distress [10].

Identification of factors predictive of psychological distress is important, as this can help clinicians determine who requires close monitoring to prevent significant psychological distress. We first investigated psychological distress in patients with primary bone and soft tissue tumors with no metastasis and no history of depression and/or anxiety. In this study, we used the DIT and identified psychological distress in 21% of them. Multivariate analysis revealed that older age, female sex, intermediate or malignant tumors, and higher NRS were risk factors for psychological distress.

The Palliative Care Emphasis Program on Symptom Management and Assessment for Continuous Medical Education (PEACE) program, which is a large national project that provides an educational program about supportive care established by the Japanese Cancer Control Act, has also recommended psychological screening by DIT [27].

### 4.2. Identification of Patients at a Higher Risk of Psychological Distress in Bone and Soft Tissue Tumor Patients

It is reported that women have higher prevalence and higher mean level of psychological distress than men in general population [28,29,30]. There are several reports that gender (female) was a risk factor for psychological distress in various cancer [31,32,33,34]. Similar to previous studies, we found significantly higher levels of distress in women than in men. We also identified psychological distress more frequently in older patients. Although younger age is associated with psychological distress in several reports of cancers, Parades reported that older age is a risk factor for psychological distress in patients with malignant bone and soft tissue tumors treated with chemotherapy or surgery [12]. Patients with malignant bone and soft tissue tumors are relatively younger than patients with other malignancies, which may explain this observation.

Psychological distress was identified in 32% of patients with malignant soft tissue and bone tumors, and in only 15% of patients with soft tissue and bone benign tumors. Pre-operative patients with malignant soft tissue and bone tumors are faced with the threat of the disease itself (aggressiveness for local recurrence and distant metastasis, symptoms such as pain and physical and functional impairments), and its treatment (the risk of permanently restricted mobility and reduced physical functioning after wide resection and risk of limb amputation in some patients) [10,12,13,17,18].

Pain was also a risk factor for psychological distress in patients with primary bone and soft tissue tumors. Furthermore, pain levels were also correlated with psychological distress. Pain is one of the most significant risk factors for psychological distress in cancer patients [35]. Pain negatively influences mental well-being and quality of life and is associated with decreased levels of social activities and social support [36,37]. Thus, pain should be managed properly before surgery, and if possible, at the time of presentation.

In this study, psychological distress was identified in 10 of 21 patients with malignant bone tumors, and in 24 of 84 patients with malignant soft tissue tumors. Multivariate analysis revealed that not receiving neoadjuvant chemotherapy was the only risk factor for psychological distress. Although chemotherapy is frequently accompanied by nausea, dullness, and other adverse events that can increase psychological distress, chemotherapy may also relieve pain and other local symptoms by shrinking the tumor, which can decrease psychological distress [38].

To the best of our knowledge, no study has assessed psychological distress in patients with benign bone and soft tissue tumors. In this study, we identified psychological distress in 21% of them. Among soft tissue tumors, psychological distress was identified in 26% of patients with schwannomas, which was higher than that in patients with other benign soft tissue tumors and as high as that in those with malignant soft tissue tumors. Multivariable analysis revealed that older age and higher NRS (pain during motion) were risk factors for psychological distress. Patients with benign soft tissue and bone tumors who have pain are likely to undergo surgical excision. Thus, NRS could be related to psychological distress.

With DIT, we identified 64 patients (21%) with psychological distress, a lower number than identified by DT (95 patients (32%)). By adding DIT-I, which identifies how much impact the distress has on their daily life activity, we narrowed down the patients. Although we asked whether they hoped for an intervention by a psychiatrist, only two patients hoped to consult with a psychiatrist.

Clover reported that only 29% of patients with psychological distress seek help for their distress, and in particular, younger patients and women were more likely to decline help [39]. He reported that the three main reasons for declining help with distress were a preference to self-manage, already receiving help elsewhere, and distress not severe enough to warrant intervention. Kim reported that Asians and Asian Americans are more reluctant to ask for support from others than are European Americans because they fear the risk of disturbing relationships with each other [40]. Japanese people also tend to underutiliize mental health service than other developed countries [41]. Whether cancer patients seek help for their distress, there are differences between racial, cultural, and social contexts so qualitative research and subsequent interventions for overcoming these barriers are required to obtain the most benefit from distress screening programs.

### 4.3. Limitations and Future Research

This study has some limitations. First, because bone and soft tissue tumors are various and rare, the number of patients with each type of tumor was small, which limited our ability to compare the rate of psychological distress in each tumor type. Thus, in a larger series of patients, other factors may be discovered, such as important risk factors for psychological distress. Another limitation was that the cut-off score for screening for psychological distress is not specific for bone and soft tissue tumors. In this study, we used the cut-off scores (DIT-D ≥ 4 and DIT-I ≥ 3) that were previously recommended [3]. Itani reported that DIT-D ≥ 4 and DIT-I ≥ 2 are reliable cut-off scores for screening for emotional distress among gynecological cancer patients by using HADS as the gold standard [6]. However, no gold standard cut-off score has been established for bone and soft tissue tumors, and this should be investigated in the future.

## 5. Conclusions

In conclusion, using the DIT, psychological distress was identified in 64 patients (21%) with primary bone and soft tissue tumors. Psychological distress was identified in 32%, 18%, and 15% of patients with malignant, intermediate, and benign bone and soft tissue tumors, respectively. Careful attention to psychological distress is needed, especially for female patients, older patients, and those with malignant soft or bone tissue tumors who have more than moderate pain. As cancer patients tend to decline interventions, qualitative research and subsequent interventions for overcoming these barriers are required.

## Figures and Tables

**Table 1:** Patients’ characteristics.

Characteristic		Median (Range)	Number	%
Age (years)	Median (range)	53 (18–87)		
Sex	Male		172	58%
	Female		126	42%
Body mass index (kg/m^2^)	Median (range)	24 (13–44)		
Type of tumor	Bone		54	18%
	Soft tissue		244	82%
Malignancy	Malignant		105	35%
	Intermediate		32	11%
	Benign		161	54%
Anatomical location	Upper limb		99	33%
	Lower limb		135	45%
	Trunk		64	22%
Pain: In motion (NRS)	Median (range)	2.6 (0–10)		
Barthel Index	Median (range)	98.3 (35–100)		
Performance status	0		264	88%
	1		30	10%
	2		2	1%
	3		2	1%
Current employment	Full-time		169	57%
	Part-time		11	4%
	Retired		80	27%
	Other		38	13%
Smoking	Yes		57	19%
	No		241	81%
Drinking	Yes		93	31%
	No		205	69%
Marital status	Married		227	76%
	Divorced/never married		71	24%
Housemate	Yes		256	86%
	No		42	14%
Children	Yes		207	69%
	No		91	31%

**Table 2:** Assessment of psychological distress in patients with bone tumors.

Variables	Histology	Presence of Psychological Distress	Abesence of Psychological Distress	Number
		(*n* = 15)	(*n* = 39)	(*n* = 54)
Malignant (*n* = 21)	Osteosarcoma	4	4	8
	Chondrosarcoma (G2)	4	6	10
	Ewing sarcoma		1	1
	Clear cell chondrosarcoma	1		1
	Chordoma	1		1
Intermediate (*n* = 15)	Giant cell tumor	2	3	5
	Aneurysmal bone cyst	1	3	4
	Chondroblastoma	1	3	4
	Chondrosarcoma (G1)	1	1	2
Benign (*n* = 18)	Exostosis		10	10
	Enchondroma		3	3
	Osteoid osteoma		3	3
	Intraosseous lipoma		1	1
	Bizarre parosteal osteochondromatous proliferation		1	1

**Table 3:** Assessment of psychological distress in patients with soft tissue tumors.

Variables	Histology	Presence of Psychological Distress	Abesence of Psychological Distress	Number
		(*n* = 49)	(*n* = 195)	(*n* = 244)
Malignant (*n* = 84)	Liposarcoma	7	19	26
	Undifferentiated sarcoma	7	15	22
	Myxofibrosarcoma	4	14	18
	Fibrosarcoma	1	2	3
	Synovial sarcoma	1	2	3
	Epithelioid sarcoma	2		2
	Dermatofibrosarcoma protueberans		2	2
	Leiomyosarcoma		1	1
	Extraosseous osteosarcoma	1	1	2
	Malignant glomus tumor	1		1
	Clear cell sarcoma		1	1
	Malignant peripheral nerve sheath tumor		1	1
	Other		2	2
Intermediate (*n* = 16)	Well differentiated liposarcoma	1	8	9
	Solitary fibrous tumor		5	5
	Other		2	2
Benign (*n* = 144)	Lipoma	6	45	51
	Schwannoma	11	31	42
	Tenosynovial giant cell tumor	4	28	32
	Neurofibroma	1	3	4
	Fibroma	1	4	5
	Nodular fasciitis		1	1
	Leiomyoma	1		1
	Hemangioma		1	1
	Myxoma		1	1
	Glomus tumor		1	1
	Other		5	5

**Table 4:** Univariate and multivariable logistic regression of risk factors related to psychological distress in all patients.

Variables	Presence of Psychological Distress	Abesence of Psychological Distress	Univariate Analysis	Multiple Logistic Regression Analysis	*p*-Value
	(*n* = 64)	(*n* = 234)	*p*-Value	OR (95% CI)	
Age, years, median (range)	57 (26–82)	52 (18–87)	<0.01 **	1.01 (1.00–1.03)	<0.05 *
Sex					
Female	34 (53%)	92 (39%)	<0.05 *	2.00 (1.08–3.73) *	<0.05 *
Male	30 (47%)	142 (61%)			
BMI, kg/m^2^ median (range)	24 (18–34)	24 (14–44)	0.31		
Type of tumor					
Bone tumor	15 (23%)	39 (21%)	0.26		
Soft tissue tumor	49 (77%)	195 (79%)			
Malignancy					
Benin	24 (38%)	137 (58%)	<0.01 **	2.15 (1.13–4.08)	<0.05 *
Intermediate/Malignant	40 (62%)	97 (42%)			
Anatomical location					
Upper limb	19 (30%)	80 (34%)	0.58		
Lower limb	33 (52%)	102 (44%)	0.15		
Trunk	12 (19%)	52 (22%)	0.29		
Pain: In motion, median (range)	3.8 (0–10)	2.2 (0–10)	<0.01 **	1.14 (1.01–1.29) *	<0.05 *
Performance status, median (range)	0.3 (0–4)	0.1 (0–4)	<0.01 **	1.20 (0.64–2.26)	0.57
Barthel Index (range)	96 (0–100)	99 (0–100)	<0.01 **	1.07 (1.00–1.15)	<0.05 *
Current employment					
Full-time	31 (48%)	138 (59%)			
Part-time	2 (3%)	9 (4%)	0.71		
Retired	13 (20%)	25 (11%)	0.35		
Other	18 (28%)	62 (26%)	0.47		
Pre-operative chemotherapy					
Yes	2 (3%)	18 (8%)	0.15		
No	62 (97%)	216 (92%)			
Pre-operative radiotherapy					
Yes	1 (2%)	2 (1%)	0.61		
No	63 (98%)	232 (99%)			
Smoking					
Yes	22 (34%)	98 (42%)	0.31		
No	42 (66%)	136 (58%)			
Drinking					
Yes	24 (38%)	107 (46%)	0.25		
No	40 (62%)	127 (54%)			
Marital status					
Married/divorced	52 (81%)	175 (75%)	0.27		
Never married	12 (19%)	59 (25%)			
Housemate					
Yes	54 (84%)	202 (86%)	0.66		
No	10 (16%)	32 (14%)			
Children					
Yes	50 (78%)	157 (67%)	0.09		
No	14 (22%)	77 (33%)			

BMI = Body Mass Index; CI = confidence interval; OR = odds ratio. * *p* < 0.05, ** *p* < 0.01.

**Table 5:** Univariate and multivariable logistic regression of risk factors related to psychological distress in patients with malignant bone and soft tissue tumors.

Variables	Presence of Psychological Distress	Abesence of Psychological Distress	Univariate Analysis	Multiple Logistic Regression Analysis	*p*-Value
	(*n* = 34)	(*n* = 71)	*p*-Value	OR (95% CI)	
Age, years, median (range)	58 (24–82)	57 (18–87)	0.37		
Sex					
Female	19 (56%)	21 (30%)	<0.01 **	1.37 (0.49–3.81)	0.54
Male	15 (44%)	50 (71%)			
BMI, kg/m^2^, median (range)	23 (18–33)	24 (13–38)	0.26		
Malignancy					
Bone tumor	10 (29%)	11 (16%)	0.12		
Soft tissue tumor	24 (71%)	60 (84%)			
Anatomical location					
Upper limb	8 (24%)	15 (21%)	0.90		
Lower limb	17 (50%)	34 (49%)			
Trunk	9 (26%)	22 (30%)			
Pain: In motion, median (range)	2.7 (0–10)	2.6 (0–10)	0.49		
Performance status, median (range)	0.4 (0–4)	0.3 (0–4)	0.29		
Barthel Index (range)	97 (0–100)	95 (0–100)	0.08		
Current employment					
Full-time	15 (44%)	37 (53%)			
Part-time	0 (0%)	1 (1%)	0.48		
Retired	10 (29%)	15 (21%)	0.53		
Other	9 (27%)	18 (25%)	0.13		
Pre-operative chemotherapy					
Yes	2 (6%)	17 (26%)	<0.05 *	6.07 (1.22–30.1)	<0.05 *
No	32 (94%)	54 (74%)			
Pre-operative radiotherapy					
Yes	1 (3%)	2 (3%)	0.97		
No	33 (97%)	69 (97%)			
Smoking					
Yes	6 (18%)	32 (44%)	<0.01 **	2.75 (0.84–9.00)	0.10
No	28 (82%)	39 (56%)			
Drinking					
Yes	10 (29%)	37 (52%)	<0.05 *	1.85 (0.66–5.24)	0.25
No	24 (71%)	34 (48%)			
Marital status					
Married/divorced	26 (76%)	56 (78%)	0.85		
Never married	8 (24%)	15 (22%)			
Housemate					
Yes	29 (85%)	64 (90%)	0.43		
No	5 (15%)	7 (10%)			
Children					
Yes	24 (71%)	51 (71%)	0.95		
No	10 (29%)	20 (29%)			

BMI = Body Mass Index; CI = confidence interval; OR = odds ratio. * *p* < 0.05, ** *p* < 0.01.

**Table 6:** Univariate and multivariable logistic regression of risk factors related to psychological distress in patients with benign bone and soft tissue tumors.

Variables	Presence of Psychological Distress	Abesence of Psychological Distress	Univariate Analysis	Multiple Logistic Regression Analysis	*p*-Value
	(*n* = 24)	(*n* = 137)	*p*-Value	OR (95% CI)	
Age, years, median (range)	58 (35–78)	49 (18–84)	<0.01 **	1.04 (1.01–1.08)	<0.05 *
Sex					
Female	12 (50%)	58 (42%)	0.48		
Male	12 (50%)	79 (58%)			
BMI, kg/m^2^, median (range)	24 (19–34)	24 (16–44)	0.40		
Malignancy					
Bone tumor	0 (0%)	18 (13%)	0.06		
Soft tissue tumor	24 (100%)	119 (87%)			
Anatomical location					
Upper limb	8 (33%)	58 (42%)	0.44		
Lower limb	13 (54%)	55 (40%)			
Trunk	3 (13%)	24 (18%)			
Pain: In motion, median (range)	5.4 (0–10)	2.0 (0–10)	<0.01 **	1.41 (1.19–1.69)	<0.01 **
Barthel Index (range)	96 (0–100)	100 (0–100)	0.06		
Performance status, median (range)	0.13 (0–4)	0.01 (0–4)	0.19		
Current employment					
Full-time	14 (58%)	87 (64%)			
Part-time	1 (4%)	5 (4%)	0.45		
Retired	2 (8%)	6 (4%)	0.77		
Other	7 (29%)	39 (28%)	0.44		
Smoking					
Yes	12 (50%)	57 (42%)	0.44		
No	12 (50%)	80 (58%)			
Drinking					
Yes	13 (54%)	57 (42%)	0.25		
No	11 (46%)	80 (58%)			
Marital status					
Married/divorced	20 (83%)	105 (77%)	0.47		
Never married	4 (17%)	28 (23%)			
Housemate					
Yes	19 (79%)	117 (85%)	0.44		
No	5 (21%)	20 (15%)			
Children					
Yes	20 (83%)	94 (69%)	0.14		
No	4 (17%)	43 (31%)			

BMI = Body Mass Index; CI = confidence interval; OR = odds ratio. * *p* < 0.05, ** *p* < 0.01.

## Data Availability

The datasets used and analyzed during the current study are available from the corresponding author on reasonable request.
